# Association of Polymorphism of the Methyl Tetrahydrofolate Reductase (MTHFR) Gene with Anti-Seizure Medication Response in Pediatric Patients in Jeddah, Saudi Arabia

**DOI:** 10.3390/medicina58111593

**Published:** 2022-11-03

**Authors:** Reem Alyoubi, Abdullah Althomali, Rania Magadmi, Hala S. Abdel kawy, Hadiah Bassam Al Mahdi, Fatemah O. Kamel, Duaa M. Bakhshwin, Maha Jamal, Mohammed Alsieni

**Affiliations:** 1 Pediatric Department, Faculty of Medicine, King Abdulaziz University, Jeddah 21589, Saudi Arabia; 2 Pharmacology Department, Faculty of Medicine, King Abdulaziz University, Jeddah 22254, Saudi Arabia; 3 Department of Clinical Pharmacy, King Faisal Medical Complex, Ministry of Health, Taif 26724, Saudi Arabia; 4 Biological Sciences Department, Faculty of Science, King Abdulaziz University, Jeddah 21589, Saudi Arabia; 5 Princess Al Jawhara Albrahim Centre of Excellence in Research of Hereditary Disorders (PACER-HD), King Abdulaziz University, Jeddah 21589, Saudi Arabia

**Keywords:** anti-seizure medication, epilepsy, gene polymorphism, MTHFR, pediatric

## Abstract

*Background and Objectives*: Epilepsy is a chronic brain disease, with inherent and noninherent factors. Although over 20 anti-seizure medications (ASMs) are commercially available, nearly one-third of patients develop drug-resistant epilepsy. We evaluated the association between the clinical features and the methyl tetrahydrofolate (MTHFR) rs1801133 polymorphism and ASMs response among pediatric patients with epilepsy. *Materials and Methods*: This was a multicenter, retrospective, case–control study of 101 children with epilepsy and 59 healthy children in Jeddah. The MTHFR rs1801133 polymorphism was genotyped using the real-time polymerase chain reaction TaqMan Genotyping Assay. *Results*: Among the patients with epilepsy, 56 and 45 showed good and poor responses to ASMs, respectively. No significant genetic association was noted between the single-nucleotide polymorphism (SNP) rs1801133 within the MTHFR gene and the response to ASMs. However, a significant association was noted between reports of drug-induced toxicity and an increase in allele A frequencies. The MTHFR rs1801133 genotype was significantly associated with the development of electrolyte disturbance among good and poor responders to ASMs. *Conclusions*: This is the first pharmacogenetic study of MTHFR in patients with epilepsy in Saudi Arabia that found no significant association between the MTHFR SNP rs1801133 and gene susceptibility and drug responsiveness. A larger sample size is needed for testing gene polymorphisms in the future.

## 1. Introduction

Epilepsy is a chronic neurological disease of the brain, characterized by recurrent seizures and abnormal electrical depolarization of the neurons in the central nervous system (CNS), at least two unprovoked seizures, with or without a loss of consciousness, and behavioral, cognitive, and emotional alterations [1]. Globally, epilepsy is one of the important neurological disorders that threatens human longevity and leads to abnormal development. According to the World Health Organization, epilepsy affects 50 million people worldwide, and approximately 35% of patients are resistant to anti-seizure medication (ASMs) [2]. The prevalence of patients with epilepsy in Saudi Arabia is 6.54 per 1000 persons [3].

Remarkably, the prevalence of pharmaco-resistant epilepsy is 25% (16.8–34.3%) in children and 13.7% in community-based populations. However, the incidence of pharmaco-resistant epilepsy is 36.3% in clinic-based cohorts [4].

The exact pathophysiology of epilepsy is not understood. What is known is that hyperexcitability and an alteration in the synaptic transmission of brain neurons are the two cardinal features of epileptogenesis [5]. The accumulating evidence suggests that the sulfur-containing amino acid homocysteine plays a potential role in developmental disorders [6]. The main factors affecting the concentration of homocysteine in the human body are folate and vitamin B12. Changes in the plasma concentrations of folate and vitamin B12 can be achieved through the diet. However, gene polymorphisms can contribute to the metabolism of folate and vitamin B12-dependent homocysteine. This is important for methylation processes, particularly for DNA methylation [7].

The enzyme 5,10-Methylenetetrahydrofolate reductase (MTHFR) converts 5,10-methylenetetrahydrofolate into 5-methyltetrahydrofolate (5-MTHF), which is the most bioactive form of folic acid. The 5-MTHF, in turn, participates in vitamin B12-dependent remethylation of homocysteine to methionine (a sulfur-containing amino acid). Then, the methionine is converted to S-adenosylmethionine (SAMe) by methionine adenosyl transferase. SAMe is important in many transmethylation reactions, such as those of neurotransmitters, proteins, and DNA methylation [8].

Consequently, this study aimed to investigate the correlation between the genetic polymorphism of MTHFR and drug-resistant epilepsy in pediatric patients with epilepsy in Jeddah, Saudi Arabia.

## 2. Materials and Methods

### 2.1. Ethical Approval

This study was approved by the Biomedical Ethics Research Committee of the Faculty of Medicine, King Abdulaziz University, on 26 October 2020 (approval number 530-20). 

### 2.2. Subjects and Data Source

The study population consisted of 101 pediatric patients with epilepsy (68 males, 33 females) and a control group of 59 participants without the disease (39 males, 20 females). The participants were residents of Jeddah, Saudi Arabia. The pediatric patients with epilepsy were outpatients, recruited from King Abdulaziz University Hospital and Soliman Fakeeh Hospital in Jeddah.

### 2.3. Study Design

This was a hospital-based, multicenter, case–control, retrospective study of pediatric patients diagnosed with epilepsy. At the beginning, demographic and clinical data were collected from the patients’ caregivers. All the data were collected through a designed electronic data collection sheet. Each participant was assigned a unique code, rather than a patient name, to ensure patient privacy and security.

### 2.4. Patients Recruitment

The principal investigator, a pediatric neurology consultant, assessed the patients who met the inclusion criteria during their clinic visit. After the principal investigator provided an explanation of the research aim and process to the caregivers, they were asked to read and sign a written informed consent form. This was witnessed by a co-investigator.

### 2.5. Inclusion and Exclusion Criteria

The inclusion criteria were patients who had had at least two seizure attacks within 24 h, were aged between 2 and 17 years, had been receiving ASMs for at least 1 year, and had normal psychometric development, a normal neurologic examination, and normal background activity. The exclusion criteria were patients without records, unreliable seizure frequency, poor compliance with ASMs, presence of a liver disorder, or refusal of written informed consent.

**Figure 1:**
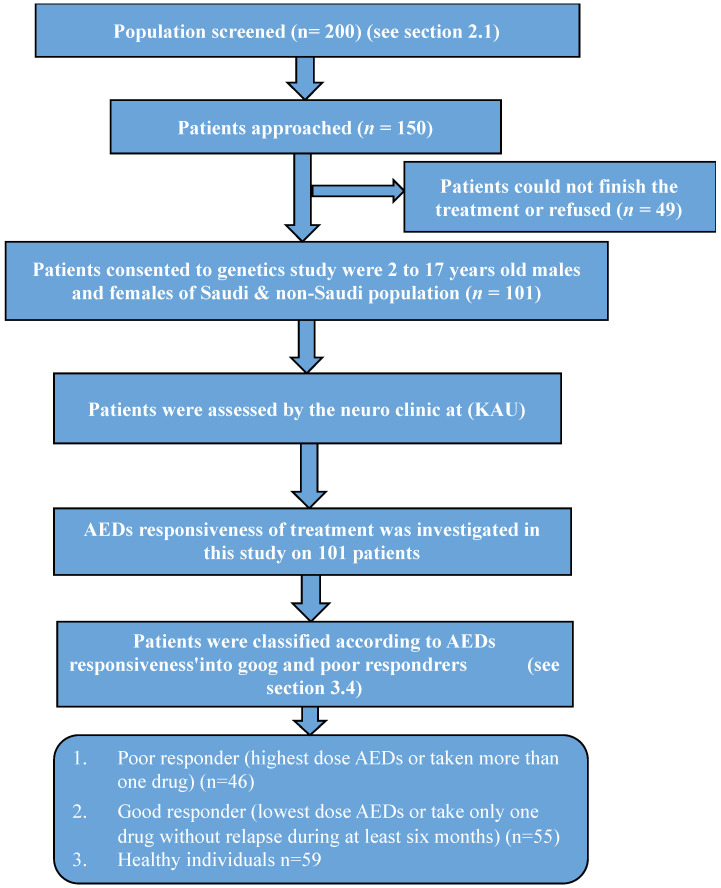
Flowchart of enrollment and analysis process.

Initially, the sample size was calculated using OpenEpi info (CDC, Atlanta). The calculated sample size was 200 patients who were screened (Figure 1). Based on the inclusion and exclusion criteria, 150 patients were approached to participate in this study. Of these, 49 patients could not finish the procedure program, for clinical reasons, or refused. Of the remaining patients, 101 agreed to be part of the study.

### 2.6. Outcome Measures

#### 2.6.1. Clinical Outcomes

An electronic data collection sheet was designed. These sheets were used to collect demographic and clinical data for the 101 patients. The demographic data included age and nationality. The family history of patients with epilepsy was also obtained. This allowed the identification of the subset of patients with epilepsy who may be influenced by genetic factors.

The clinical data included were the age at diagnosis, disease duration, and type of the current ASM. The patients were classified into good or poor responders, based on their responsiveness to ASMs. The diagnosis of epilepsy was based on the classification system for seizure type provided by the International League Against Epilepsy (ILAE) [9].

The definition used for good drug responders was, “The patients were considered as drug-responsive if they did not have any type of seizures for at least 1 year during ASM treatment.” [10,11]. The definition used for poor drug responders was that set by the ILAE: “patients were considered as drug-resistant to epilepsy if adequate trials of two tolerated and appropriately used ASM schedules (whether monotherapy or combination therapy) failed to achieve sustained seizure freedom” [11].

#### 2.6.2. Laboratory Outcomes

At the end of the visit, a 3–5-mL blood sample was taken from each participant for laboratory and genetic analyses. Serum was prepared from each sample and analyzed for vitamin B12 concentration using the Human Vitamin B12 Elisa Kit (Catalog Number: MBS729208) following the manual instruction [7].

### 2.7. SNP Selection and Genotyping

#### 2.7.1. SNP Selection

The MTHFR gene was investigated in this study. The single-nucleotide polymorphism (SNP; rs180133) was selected from the National Center for Biotechnology Information (http://www.ncbi.nlm.nih.gov/SNP/; accessed on 2 November 2022), Ensembl database (http://www.ensembl.org/index.html; accessed on 2 November 2022), and Applied Biosystems SNP database (http://www.appliedbiosystems.com; accessed on 2 November 2022). The gene, SNP ID, and location on the chromosome are shown in Table 1.

#### 2.7.2. SNP Genotyping

A peripheral blood sample of 200 μL was used. The genomic DNA was purified using the QIAamp DNA Mini Kit (Catalog #51306, Qiagen, Alameda, CA, USA). The DNA concentration (ng/mL), quality, and quantity were measured using the NanoDrop™ 2000c Spectrophotometer (Thermo Fisher Scientific, Waltham, MA, USA).

The samples that met the quantitative requirements for this study were analyzed by using the real-time polymerase chain reaction (PCR) for SNP genotyping of the missense polymorphism, G>A rs1801133. The assay was performed using QuantStudio 3 Real-Time PCR with TaqMan (Applied Biosystems International, Foster City, CA, USA). The genotyping assay (C___1202883_20) comprised allele G labeled with VIC™ dye (green fluorophore) and allele A labeled with FAM™ dye (blue fluorophore) and used the TaqMan Genotyping Master Mix, according to the manufacturer’s instructions.

Finally, the samples were analyzed by QuantStudio 3 software v1.5.1 and TaqMan Genotyper Software (Thermo Fisher Scientific, Waltham, MA, USA) for amplification and allelic discrimination.

#### 2.7.3. Sanger Sequencing

Sanger sequencing was used to validate and check the accuracy and reproducibility of the SNP genotyping assay. Prior to sequencing, PCR amplification of 30 random DNA samples from the patients and controls was performed using the Veriti™ 96-Well Thermal Cycler, GoTaq Green Master Mix (Cat# M7122, Promega Corporation, Madison, WI, USA), and the following primers: F: 5′-TCCCTGTGGTCTCTTCATCC-3′; R: 5′-CTGGGAAGAACTCAGCGAAC-3′. The PCR products were electrophoresed on an agarose gel and then purified using the QIAquick PCR Purification Kit (Cat# 28104, Qiagen, Alameda, CA, USA). The cycle sequencing PCR reaction was performed using the BigDye Terminator v3.1 Cycle Sequencing Kit (Life Technologies, Waltham, MA, USA) and sequencing was performed using the SeqStudio Genetic Analyzer (Life Technologies). The Bioedit software version 6 (Carlsbad, CA, USA) was used for the sequence alignment and identification of the variants.

### 2.8. Statistical Analyses

The demographic, clinical, and laboratory data of the patients are presented using descriptive statistics. The frequencies and percentages are reported for the categorical variables. The continuous data are reported as the mean ± standard deviation. The differences between the groups were analyzed using Pearson’s chi-squared test for the categorical variables and the t-test for the continuous outcome variables. Significance (*p*-value) was set at 0.05. The statistical analyses were performed using the Social Sciences Statistical Package (SPSS) software version 21 (IBM, Armonk, NY, USA).

For the genotyping statistical analyses, the Hardy–Weinberg equilibrium (HWE) was used to determine whether the individual variants were in HWE at each locus in each population. Pearson’s standard chi-squared test, odds ratios, and 95% confidence intervals were calculated. A *p*-value < 0.05 was considered significant in the allele and genotyping frequencies.

## 3. Results

### 3.1. Demographic and Clinical Characteristics of Participants

The demographic characteristics of all the participants are shown in Table 2. The study group consisted of 101 patients with epilepsy and 59 control participants. The mean age for the patient group was 7.3 ± 4.1 years vs. 8.8 ± 3.2 years for the control group. In each group, approximately one-third of the participants were females and approximately two-thirds were of Saudi nationality. In addition, one-third of the participants in each group had first-degree parental consanguinity. The majority of participants in both groups had no family history of epilepsy.

### 3.2. MTHFR Genotype Distribution Polymorphism and Allele Frequencies

All the controls were in HWE at the MTHFR rs1801133 G>A (chi-square = 0.26, *p* = 0.61), while chi-square = 0.48, *p* = 0.49 for cases. The HWE for all samples was as follows: chi-square = 0.7, *p* = 0.4.

**Figure 2:**
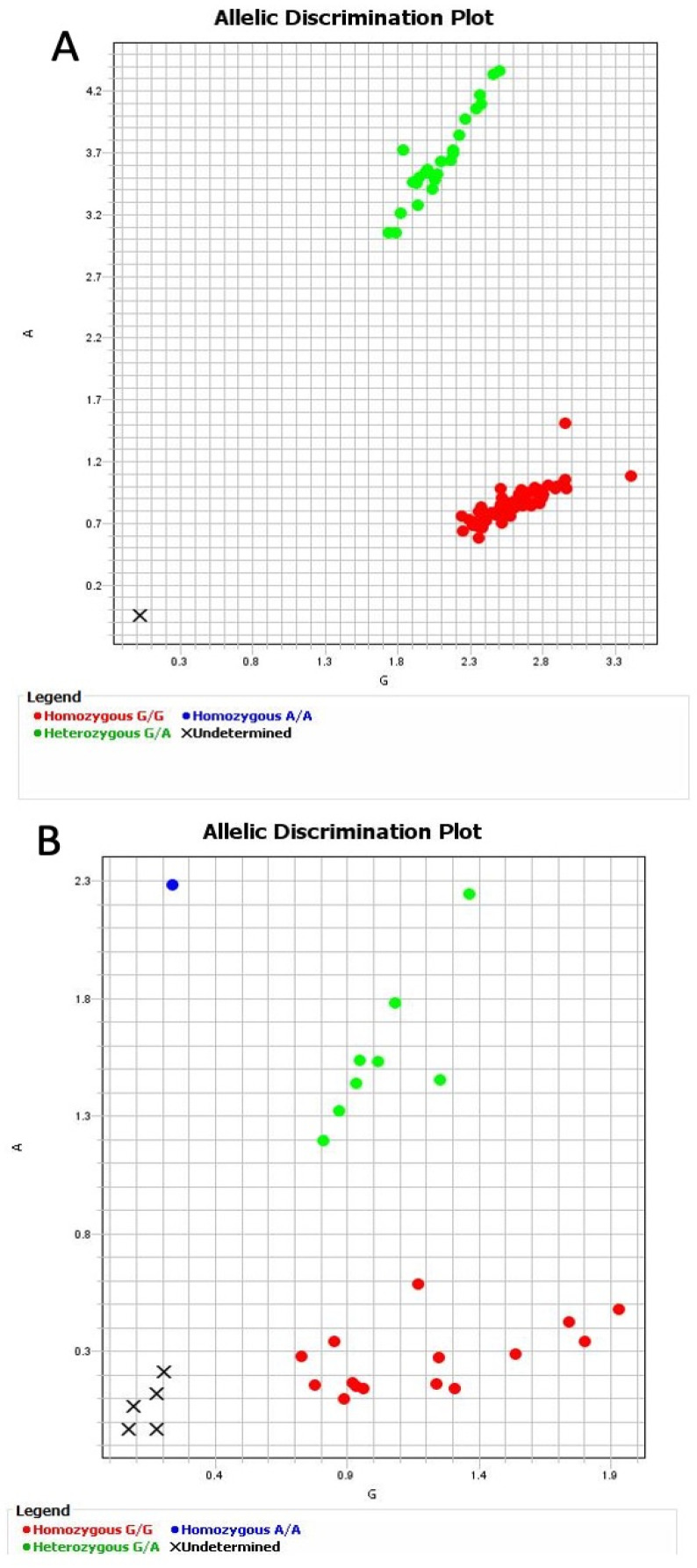
Allelic discrimination plot for rs1801133 from random samples: (**A**) control samples; (**B**) patient samples.

**Figure 3:**
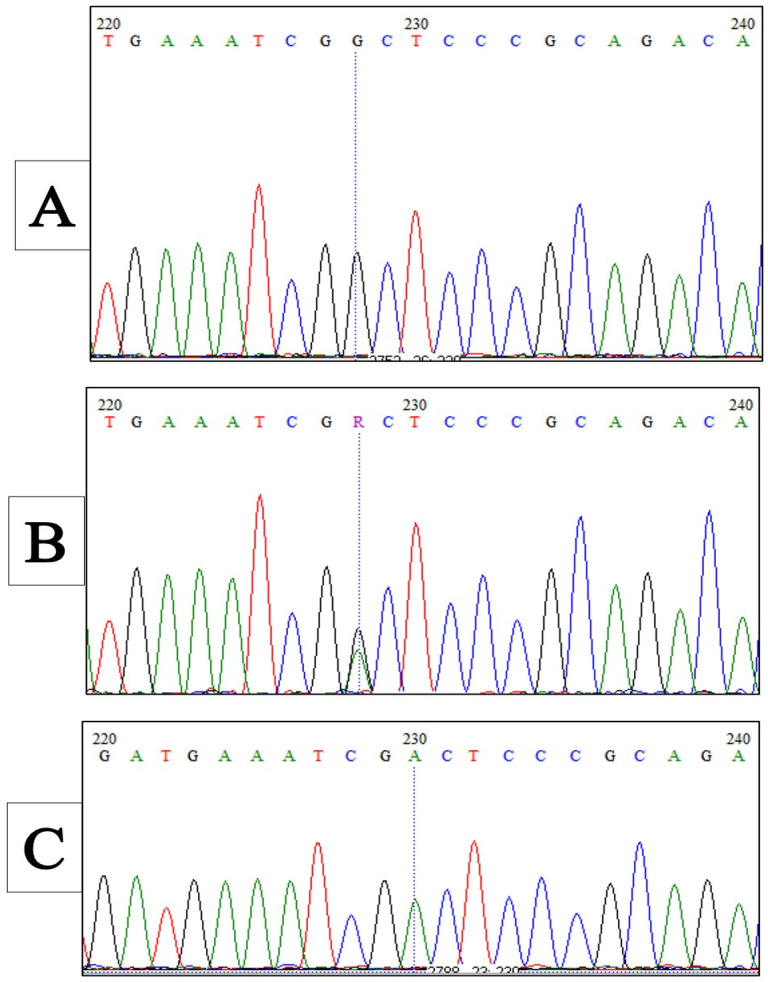
Electropherograms for rs1801133 validation: (**A**) homozygous G; (**B**) heterozygous G/A; (**C**) homozygous A.

A representative plot for the random, control (A), and patient (B) samples is presented in Figure 2 and was confirmed by electropherograms and Sanger sequencing, as shown in Figure 3.

As shown in Table 3, in the patient group (rs1801133), 74.3% of the patients were homozygous dominant, 24.6% were heterozygous, and 1% were homozygous recessive. The allele frequencies of A and G were 13.3% and 86.7% in the patient group. In the comparison between the healthy controls and the patients with epilepsy, no genetic association was found for the studied SNP of MTHFR (rs1801133).

### 3.3. Association of MTHFR SNP Genotypes with Patient Response to ASMs

Based on the ASM classification, approximately half the patients (54.5%) showed a good response to ASM; the other half (45.5%) were poor responders. The mutant variant (AA) appeared in the poor responders’ group only, as shown in Table 4. However, there was no significant difference between the two groups in all the genotype frequencies.

Next, the dominant and recessive models were used to assess the association between carrying the A allele or G allele and the patient’s response to the ASM. The co-dominant model was used to assess the association between heterozygotes and the patient’s response to the ASMs, as shown in Table 5. In all the models tested, there was no significant association between the patient’s response to ASMs and the MTHFR gene polymorphism.

### 3.4. Association of MTHFR SNP Genotypes with Vitamin B12 Level in Patients with Epilepsy

As MTHFR is involved in vitamin B12 metabolism, the mean vitamin B12 concentrations were compared among all the MTHFR polymorphisms (GG, GA, and AA) in the patients with epilepsy, as shown in Table 6. The statistical analyses did not reveal any differences.

Data were analyzed using the chi-squared test.3.5 MTHFR genotype rs1801133 and electrolyte disturbances caused by ASMs. The clinical parameters of the patients with epilepsy showed that almost 90% of poor responders had electrolyte disturbances (41 of 46 poor responders), whereas only 40% of good responders had electrolyte disturbances (22 of 55 poor responders). As a result of these significant differences (*p* < 0.0001), the MTHFR genotype was compared between the patients with and without electrolyte disturbances. The results showed that the dominant and recessive MTHFR genotype polymorphism rs1801133 had a statistically significant association with the model of electrolyte disturbance (*p* = 0.022 and *p* = 0.04; Table 7).

### 3.5. MTHFR Genotype rs1801133 and Electrolyte Disturbances among Good and Poor ASM Responders

The MTHFR rs1801133 genotype was significantly associated with the incidence of electrolyte disturbance among good and poor ASM responders (*p* = 0.011; Table 8).

## 4. Discussion

Epilepsy is a common disorder of the CNS that leads to serious morbidities and a significant death rate [12]. It commonly presents as recurring, unprovoked convulsions caused by excessive and hypersynchronous electrical activity in the brain. Children are more prone to the development of epilepsy, especially after brain insults; approximately 50% of patients with epilepsy have their first seizure during childhood, and 50% of children with epilepsy have their first seizure during infancy (aged younger than 1 year) [13]. The immature brain of a child is more susceptible to developing seizures than the mature brain of an adult, and these seizures are commonly more severe and precipitated by triggers that are different from those of the mature brain. The physiologically immature ion homeostasis and other developmental characteristics, along with the aforementioned factors, make treating childhood epilepsy challenging [13,14].

The response to medication varies greatly between individuals [15]. The concept of pharmaco-resistance is a well-known problem in clinical practice. Uncontrolled seizures or epilepsy are strongly related to pharmaco-resistant epilepsy. The failure to achieve seizure control with the first or second drug trial of ASMs given at the appropriate daily dosage, despite the fact that these drugs possess different modes of action, is termed pharmaco-resistance [11]. Despite the presence of several ASMs, including carbamazepine, phenytoin, valproate, and gabapentin, approximately one-third of patients with epilepsy develop pharmaco-resistance to the available ASMs [2]. Moreover, ASM pharmaco-resistance is one of the major causes of mortality, with a high prevalence in developing countries [16]. Indeed, pharmaco-resistant epilepsy is associated with a fivefold higher mortality rate than that in the general population. Many factors can affect the patient’s response to the ASM. The early prediction of a patient’s response to the ASM could save their time and health. Thus, this study aimed to investigate the correlation between the genetic polymorphism of the MTHFR enzyme and drug-resistant epilepsy in pediatric patients with epilepsy in Jeddah, Saudi Arabia.

Among the sample population of this study, 45% of the participants showed poor responses to ASMs, based on the ILAE criteria. This percentage is slightly higher than those reported in the previous works [17,18]; however, a more recent study performed in another Arab population (of pediatric patients with epilepsy from Jordan) found a percentage of drug resistance similar to that in this study [19]. Moreover, two previous studies of Indian patients reported that 44–46% of patients were poor responders [20,21]. Consequently, this similarity in the prevalence of ASM drug resistance among the same ethnicity highlights the importance of pharmacogenetics in ethnic differences in response to ASMs.

Some ASMs, such as phenytoin and carbamazepine, directly affect the activity of variant liver enzymes. Liver enzyme induction can cause the depletion of cofactors, such as vitamin B12, leading to the observed elevation in the homocysteine level [7,22]. The methylated forms of vitamin B12 can cause problems related to MTHFR gene handling. Nevertheless, a hereditary predisposition to vitamin B12 deficiency was observed in different studies [23]. For example, the results from a previous study in Jordan showed a significant correlation between 677CT of the MTHFR gene mutation and a vitamin B12 deficiency in their population [24].

Given the above evidence, vitamin B12 was evaluated in this study. All patients with epilepsy have, to some extent, a vitamin B12 deficiency, but no statistical significance was observed when B12 levels were compared between good and poor responders. Therefore, this result indicated that vitamin B12 alone could not predict a patient’s response to ASM. Therefore, testing a patient’s genetic profile for polymorphisms is the logical next step in this project.

Heredity is one of the causes of pharmaco-resistance [16]. Drug targets are one of the many theories that have emerged to account for such resistance; this suggests that pharmacodynamics or kinetic impairment is caused by genetic alterations at the drug target sites. As indicated previously, the development of ADE-pharmaco-resistance depends on several factors, such as genetic differences between individual factors [25].

Regarding the pharmacogenetic drug response, it is difficult to study the heritable component of phenotypic variance using family pedigree studies, for example, when investigating disease susceptibility, because it is extremely rare to find family members with the same disease who are receiving the same regimen and have the same well-defined drug response. Therefore, studying the pharmacogenetic drug response by using gene association studies is more practical [26]. This highlights the need for research such as the current study.

In a previous study, the cohort from Jordan was used to investigate the genetic association of MTHFR gene polymorphisms with susceptibility to the development of epilepsy and response to treatment [19]. The rs1801133 SNP within the MTHFR gene was found to be associated with epilepsy. The frequency of the rare homogenous genotype of this SNP in healthy controls was 10.7%, which was the highest in the Middle East [27], followed by 7.5% in Turkey [28], and less than 1% in sub-Saharan Africa [27,29], but almost identical to that reported in Japan (10.2%) [29]. To date, no data have been published on its frequency in the Saudi Arabian population. Therefore, this was analyzed in this study.

Unlike the abovementioned studies, rs1801133 in this study was not associated with epilepsy and responsiveness. The differences in the association with MTHFR polymorphisms between this study and the Jordanian cohort study [19] could be attributed to differences in the underlying genetic stratification between both populations. Therefore, the role of the MTHFR polymorphism in decreasing the ASM response should be verified and replicated in larger cohorts. The pharmacodynamic interactions of ASMs are not well characterized, but understanding them may help to develop a more rationalized approach to the pharmacotherapy of epilepsy.

In contrast, interestingly, although the MTHFR polymorphism did not show a significant association with the ASM response, there was a significant association between the MTHFR polymorphism and a history of toxicity among the study population. However, this observation should be studied in more depth and detail.

Overall, this study tried to expand the choice of different treatment options. The early identification of individuals who are sensitive or resistant to ASMs, and therefore require lower or higher ASM doses, respectively, should reduce the risk of seizure attack events associated with inappropriate doses.

As the first pharmacogenetic study of its kind, there are some limitations to this study. First, the SNP and gene selected for analyses in our study were restricted to the reported literature (for example, there is little information about this gene and its effects on susceptibility and responsiveness to treatment), which could help predict the susceptibility of epilepsy and drug responsiveness. Second, the sample size was relatively small, and the number of patients with the AA variant was limited in this study. Third, there should be an evaluation of the effectiveness of ASMs, and the subgroup of patients with epilepsy should be defined. All these limitations require additional improvements in future investigations.

Further new polymorphisms and haplotypes are required to study the effects of combining different loci of MTHFR genes with larger cohorts to validate the association. The exploration of other genes (sodium and calcium ion channel-encoded genes) must be involved in the response to ASMs, in the context of Saudi Arabian patients with epilepsy, in the hope of finding more mutations among the different ASM pharmacodynamic and pharmacokinetic pathways affected by this gene. Finally, it will help in the development of dosing algorithms that accurately reflect the genetic diversity that explains the dosage requirements in the Saudi Arabian population.

## 5. Conclusions

To the best of our knowledge, this is the first pharmacogenetic association study of MTHFR among patients with pediatric epilepsy in Saudi Arabia using QuantStudio 3 Real-Time PCR with the TaqMan Genotyping Assay for genotyping one SNP within this gene. This pharmacogenetic study failed to show an association between SNP rs1801133 within the MTHFR gene with the susceptibility to epilepsy and ASM response among pediatric patients with epilepsy in Jeddah.

Until now, few studies have explored the role of pharmacogenetics in ASM response in Saudi Arabia. Further studies are required to identify other genetic factors that contribute to genetic susceptibility and treatment outcomes, and to improve epilepsy treatment efficacy and safety. An investigation of the contribution of multiple genes is necessary to understand the effects of the genetic makeup on the response to ASMs. Future studies may also be directed toward the use of haplotype analyses and should include a large cohort with more specific clinical phenotypic data.

## Figures and Tables

**Table 1:** SNP ID, position, and genotyping data based on the whole cohort (N = 160).

Gene	SNP ID	SNP	Chr Position	SNP Type
MTHFR	rs1801133	G > A	chr1:11796321	Missense

**Table 2:** Demographic and clinical characteristics of participants (N = 160).

Characteristic N (%)		Control (N = 59)	Patient (N = 101)
Age (years), mean (SD)		8.8 (3.2)	7.3 (4.1)
Gender	Male	39 (66.1)	68 (67.3)
	Female	20 (33.9)	33 (32.7)
Nationality	Saudi	41 (69.5)	66 (65.3)
	Non-Saudi	18 (30.5)	35 (34.7)
Parental consanguinity	First-degree	19 (32.2)	33 (32.7)
	Not first-degree	9 (15.2)	13 (12.8)
	Not relatives	31 (52.6)	55 (54.5)
Family history of epilepsy	Yes	3 (5.1)	7 (6.9)
	No	56 (94.9)	94 (93.1)

Data are presented as the mean (SD) or as number (%).

**Table 3:** Genotype distributions of MTHFR and allele frequencies among all participants.

SNP	Control N (%)	Patients N (%)	Adjusted OR (95% CI)	*p*-Value
rs1801133	N = 59	N = 101		
GG	41 (69.5)	75 (74.3)	1	0.78
GA	17 (28.8)	25 (24.6)	0.8 (0.39–8.97)	
AA	1 (1.7)	1 (1)	0.55 (0.03–8.97)	
G	99 (83.9)	175 (86.7)	0.804 (0.432–1.49)	0.51
A (MAF)	19 (16.1)	27 (13.3)		

Data are presented as the number of patients (N) and percentage (%). Data were analyzed using the chi-squared test; OR, odds ratio; CI, confidence interval.

**Table 4:** Genotype distributions of MTHFR and allele frequencies among good and poor ASM responders.

Genotype rs1801133	Good Responders N (%)	Poor Responders N (%)	Adjusted OR (95% CI)	*p*-Value
	N = 55 (54.46)	N = 46 (45.54)		
GG	43 (78.2)	32 (69.6)	1	0.33
GA	12 (21.8)	13 (28.3)	1.46 (0.59–3.61)	
AA	0 (0)	1 (2.1)	NA	
G	98 (89.1)	77 (83.7)	1.59 (0.73–3.7)	0.26
A (MAF)	12 (10.9)	15 (16.3)		

Data are presented as the number of patients (N) and percentage (%). Data were analyzed using the chi-squared test. NA, not applicable; OR, odds ratio; CI, confidence interval. ORs were estimated by logistic regression analyses after adjustment for regimen.

**Table 5:** Association between MTHFR polymorphism and patient’s response to ASMs.

Model	Genotype rs1801133	Good Responders N (%)	Poor Responders N (%)	Adjusted OR (95% CI)	*p*-Value
		N = 55 (54.46)	N = 46 (45.54)		
Dominant	GG	43 (78.2)	32 (69.6)	1	0.32
	GA + AA	12 (21.8)	14 (30.4)	1.57 (0.46–3.84)	
Recessive	GG + GA	55 (100)	45 (97.9)	1	0.21
	AA	0 (0)	1 (2.1)	NA	
Co-dominant	GG + AA	43 (78.2)	33 (71.7)	1	0.46
	GA	12 (21.8)	13 (28.3)	1.41 (0.57–3.49)	

Data are presented as the number of patients (N) and percentage. Data were analyzed using the chi-squared test. NA, not applicable; OR, odds ratio; CI, confidence interval. ORs were estimated by logistic regression analyses after adjustment for regimen.

**Table 6:** The mean vitamin B12 level by MTHFR genotype.

Genotype rs1801133	Vitamin B12 Mean (SD)	*p*-Value
GG	82.94 (17.78)	0.64
GA	79.29 (18.34)	
AA	78	

Data are presented as the mean (SD).

**Table 7:** MTHFR genotype rs1801133 and electrolyte disturbances caused by ASMs.

Model	Genotype rs1801133	Electrolyte Disturbance	No Electrolyte Disturbance	Adjusted OR (95% CI)	*p*-Value
		N = 63 (62.38)	N = 38 (37.62)		
Dominant	GG	50 (79.4)	25 (65.8)	1	0.022 *
	GA + AA	13 (20.6)	13 (34.2)	0.27 (0.09–0.87)	
Recessive	GG + GA	63 (100)	37 (97.4)	1	0.04 *
	AA	0 (0)	1 (2.6)	NA	
Co-dominant	GG + AA	50 (79.4)	26 (68.4)	1	0.068
	GA	13 (20.6)	12 (31.6)	0.36 (0.12–1.11)	

Data are presented as the number of patients (N) and percentage. Data were analyzed using the chi-squared test. NA, not applicable; OR, odds ratio; CI, confidence interval; *, statically significant. ORs were estimated by logistic regression analyses after adjustment for regimen.

**Table 8:** Distribution of MTHFR genotype rs1801133 and electrolyte disturbances among good and poor ASM responders.

Genotype rs1801133	Electrolyte Disturbance Good Responders	Electrolyte Disturbance Poor responders	Adjusted OR (95% CI)	*p*-Value
	N = 22 (34.9)	N = 41 (65.1)		
GG	21 (95.5)	29 (70.7)	1	0.011 *
GA	1 (4.5)	12 (29.3)	8.69 (1.05–72.1)	

Data are presented as the number of patients (N) and percentage. Data were analyzed using the chi-squared test. OR, odds ratio; CI, confidence interval; *, statically significant. ORs were estimated by logistic regression analyses after adjustment for regimen.

## Data Availability

The authors confirm that the data supporting the findings of this study are available within the article. The raw data that support the findings of this study are available from the corresponding author upon reasonable request.
